# Anti‐*Helicobacter pylori* Activity of *Musa* ‘False Horn’ (Musaceae), *Syzygium aromaticum* (Myrtaceae), *Psidium guajava* (Myrtaceae), *Mangifera indica* (Anacardiaceae), and *Cymbopogon citratus* (Poaceae) and Their Potential to Prevent and Eradicate *H. pylori* Biofilms

**DOI:** 10.1155/bmri/1930477

**Published:** 2026-10-28

**Authors:** Keuna Ngounou Cyrielle Almeida, Kouitcheu Mabeku Laure Brigitte

**Affiliations:** ^1^ Medical Microbiology and Antimicrobial Substances Laboratory Biotechnology Center University of Yaoundé Yaoundé Cameroon uninet.cm

**Keywords:** antibiofilm, antimicrobial, biofilm form, *Cymbopogon citratus*, *Helicobacter pylori*, *Mangifera indica*, *Musa* ‘False Horn’, planktonic form, *Psidium guajava*, *Syzygium aromaticum*

## Abstract

**Objectives:**

The present study evaluated the antimicrobial activity of extracts from five Cameroonian medicinal plants against planktonic forms of *Helicobacter pylori* isolates and assessed their ability to inhibit biofilm formation and eradicate preformed biofilms.

**Methods:**

The antimicrobial activity of 15 extracts obtained from *Psidium guajava* leaves, *Mangifera indica* leaves, *Cymbopogon citratus* aerial parts, *Musa* ‘False Horn’ ripe fruit peelings, and *Syzygium aromaticum* flower buds was evaluated against 48 planktonic forms of *H. pylori* clinical isolates using the broth microdilution method. The inhibition of biofilm formation and eradication of preformed biofilms were evaluated using the crystal violet staining assay.

**Results:**

The hexane extract of *M. indica* leaves exhibited the strongest antibacterial activity, with minimum inhibitory concentration values ranging from 31.25 to 62.5 *μ*g/mL against 3/48 (6.25%) of the tested isolates. The lowest minimum biofilm inhibition concentration (MBIC) of 62.5 *μ*g/mL was obtained with the methanol extract of *Musa* ‘False Horn’ fruit peelings and the dichloromethane extract of *P. guajava* leaves. The lowest minimum biofilm eradication concentration (MBEC) of 125 *μ*g/mL was recorded for the hexane extract of *P. guajava* leaves and the methanol extract of *Musa* ‘False Horn’ fruit peelings.

**Conclusion:**

The investigated plant extracts exhibited antibacterial activity against planktonic *H. pylori*, whereas several extracts also demonstrated promising antibiofilm activity. These findings support further phytochemical characterization, toxicity evaluation, and in vivo studies to investigate their potential as complementary therapeutic agents against *H. pylori* infections.

## 1. Introduction

Antimicrobial resistance (AMR) has emerged as one of the most significant global public health challenges, affecting both human and animal populations [1]. Recent global estimates indicate that bacterial antimicrobial‐resistant infections were directly responsible for approximately 1.14 million deaths worldwide in 2021. Without effective interventions, this figure is projected to rise to 1.91 million directly attributable deaths annually by 2050 [2]. Among the various mechanisms contributing to AMR, biofilm formation plays a crucial role by enabling microorganisms to survive antimicrobial treatment more effectively than their free‐living (planktonic) counterparts [3–5]. Biofilms are highly organized microbial communities embedded within a self‐produced extracellular matrix composed of polysaccharides, proteins, and other biomolecules. This matrix facilitates microbial adhesion to surfaces, promotes the development of structurally complex microbial communities, and protects bacterial cells from environmental stress, host immune responses, and antimicrobial agents [6]. Consequently, bacteria growing within biofilms exhibit markedly greater tolerance to antimicrobial therapy than planktonic cells, contributing to persistent and recurrent infections. Several clinically important bacterial species, including *Helicobacter pylori* (*H. pylori*) possess the ability to form biofilms, thereby enhancing their persistence, pathogenicity, and resistance to conventional antimicrobial therapies [5, 7, 8].

*H. pylori* is a gram‐negative, microaerophilic bacterium that colonizes the human gastric mucosa and is implicated in the pathogenesis of chronic gastritis, peptic ulcer disease, gastric adenocarcinoma, and mucosa‐associated lymphoid tissue lymphoma [9]. Epidemiological studies indicate that more than half of the world′s population is infected with this microorganism, with prevalence rates approaching 50% in industrialized countries and up to 80% in many emerging countries [10]. Current treatment strategies rely primarily on combination antimicrobial therapy, typically consisting of a proton pump inhibitor administered with two or more antibiotics, including amoxicillin, clarithromycin, metronidazole, tetracycline, or levofloxacin. When first‐line therapy fails, second‐line treatment often includes bismuth‐containing regimens [11, 12]. However, increasing AMR, together with the remarkable genetic diversity and biofilm‐forming capacity of *H. pylori*, has contributed to declining eradication rates worldwide [13, 14]. These challenges underscore the urgent need to identify novel therapeutic agents capable of preventing biofilm formation or disrupting established biofilms while maintaining an acceptable safety profile [15, 16].

Natural products derived from medicinal plants have long served as an important source of bioactive compounds for the prevention and treatment of infectious diseases [17, 18]. Owing to their rich phytochemical composition, long history of ethnomedicinal application, and broad spectrum of biological activities, plant‐derived metabolites have attracted increasing attention as promising alternatives or adjuncts to conventional antimicrobial therapies, particularly against multidrug‐resistant and biofilm‐forming pathogens [19–21]. Recent studies have further highlighted the potential of plant extracts to interfere with bacterial adhesion, quorum sensing, extracellular polymeric substance production, and other processes involved in biofilm development, making them attractive candidates for the control of persistent bacterial infections [22, 23]. Among the medicinal plants traditionally used in Cameroon, several have demonstrated promising antibacterial activity and therefore represent potential sources of novel anti‐*H. pylori* agents. Among these, *Musa* ‘False Horn’ (Musaceae; *Mu*. ‘False Horn’), commonly known as plantain, is widely consumed as a staple food and traditionally used for wound healing and the management of infectious and noninfectious diseases [24]. Methanolic and ethanolic peel extracts have demonstrated antibacterial activity against both gram‐negative and gram‐positive bacteria with minimum inhibitory concentration (MIC) values ranging from 100 to 300 *μ*g/mL [25].

*Syzygium aromaticum* (Myrtaceae; *S. aromaticum*), commonly called clove, has long been traditionally used to manage toothache, gastrointestinal disorders, flatulence, and uterine contractions [26]. Its essential oil exhibits antibacterial activity (MIC: 23–51 *μ*g/mL), and antibiofilm activity has also been reported against *H. pylori* [27].

*Psidium guajava* (Myrtaceae; *P. guajava*) is traditionally used in the treatment of gastrointestinal disorders, diarrhea, and other infectious diseases. Leaf extracts have reportedly demonstrated antibacterial activity against several gram‐negative and gram‐positive pathogens [28].

*Mangifera indica* (Anacardiaceae; *Ma. indica*), beyond the nutritional value of its fruits, has long been traditionally used to manage gastrointestinal disorders. Its leaf extracts have demonstrated antibacterial activity against *H. pylori* isolates, with a reported MIC of 92.61 *μ*g/mL [29].

*Cymbopogon citratus* (Poaceae; *C. citratus*), commonly known as lemongrass, is widely used as a herbal tea and traditional remedy for its antiseptic, digestive, and calming properties [30]. Ethyl acetate and methanolic leaf extracts have demonstrated antibacterial activity against *H. pylori* [31].

Although the antibacterial properties of these medicinal plants have been documented, comparative studies investigating their activity against both planktonic and biofilm forms of *H. pylori* remain limited. Moreover, most previous studies have focused primarily on planktonic bacteria, with relatively few evaluating both biofilm inhibition and eradication using a large collection of clinical isolates. Since biofilm formation is recognized as a major contributor to the persistence of *H. pylori* infection and reduced susceptibility to antimicrobial therapy, identifying plant extracts capable of targeting both bacterial growth and biofilm development may contribute to the discovery of new therapeutic strategies. Therefore, the present study evaluated the antibacterial activity of 15 crude extracts from *Mu* ‘False Horn,’ *S. aromaticum*, *P. guajava*, *Ma. indica*, and *C. citratus* against planktonic and biofilm forms of 48 clinical *H. pylori* isolates, with particular emphasis on their ability to inhibit biofilm formation and eradicate pre‐established biofilms.

## 2. Material and Methods

### 2.1. Chemicals and Culture Media

Analytical‐grade methanol, hexane, and dichloromethane were used as extraction solvents. Levofloxacin and clarithromycin used as reference antibiotics for planktonic susceptibility assays (determination of MIC values) were purchased from Tokyo Chemical Industry (TCI, Tokyo, Japan). N‐acetyl‐L‐cysteine (NAC), used as reference molecule for biofilm susceptibility assays, was purchased from Thermo Fisher Scientific Industry (Alfa Aesar, United States). *p*‐Iodonitrotetrazolium chloride (INT), used as a metabolic indicator for microbial growth visualization, was purchased from Sigma‐Aldrich. Crystal violet (TCI, CO.; LTD. 6‐15‐9‐TOSHIMA, KITA‐KU, Japan) and 70% (*v*/*v*) ethanol were used for biofilm biomass quantification. Sterile phosphate‐buffered saline (PBS, pH 7.4) was used for washing and removing nonadherent planktonic cells during biofilm assays.

Microbiological culture media, including Columbia agar and brain heart infusion (BHI) broth, as well as CampyGen gas generating packs for microaerophilic atmosphere creation, were purchased from Oxoid (Basingstoke, England). Lysed horse blood, fetal bovine serum (FBS), and Vitox Supplement, used for media enrichment, were also purchased from Oxoid (Basingstoke, England).

### 2.2. Plant Material

Fresh ripe fruit peelings of *Mu*. ‘False Horn’ and leaves of *P. guajava*, *Ma. indica*, and aerial parts (leaves and pseudostems) of *C. citratus* were collected in Melen‐Yaoundé (Centre Region of Cameroon). Flower buds of *S. aromaticum* were collected in Bangou (West Region of Cameroon) from November to December 2021. The plant materials and corresponding plant parts used in this study are summarized in Table 1. The plant species were authenticated at the National Herbarium of Cameroon (HNC), where voucher specimens were deposited under the Reference Numbers 67498/HNC (*Mu.* ‘False Horn’), 45028/HNC (*P. guajava*), 57347/HNC (*Ma. indica*), 36148/HNC (*C. citratus*), and 67508/HNC (*S. aromaticum*). All raw materials were thoroughly washed with tap water to remove superficial debris, rinsed with distilled water, and uniformly spread in thin layers onto clean sheets. Drying was conducted for 14 days under indoor shade conditions at room temperature (25^°^C ± 2^°^C) in a controlled, dust‐, and insect‐free laboratory environment. The dried plant parts were then ground into fine powders using a mechanical grinder and stored in airtight containers at room temperature until extraction.

**Table 1:** Plant materials, part used, extraction solvents, and extraction yields.

Plants	Parts used	Solvents	Yield (%)
*Psidium guajava*	Leaves	DCM	5.4
		Hex	10.4
		MeOH	16.61

*Mangifera indica*	Leaves	DCM	3.9
		Hex	8.78
		MeOH	10.6

*Musa* ‘False Horn’	Ripe fruit peelings	DCM	5.58
		Hex	3.20
		MeOH	10.64

*Cymbopogon citratus*	Aerial parts (leaves and pseudostems)	DCM	1.82
		Hex	0.82
		MeOH	8.48

*Syzygium aromaticum*	Flower buds	DCM	7.50
		Hex	3.08
		MeOH	23.46

Abbreviations: DCM, dichloromethane; Hex, hexane; MeOH, methanol.

### 2.3. Microbial Strains

A total of 48 clinical isolates of *H. pylori*, previously recovered from gastric biopsy specimens collected from dyspeptic patients at Laquintinie Hospital, Douala, Cameroon, were used in this study. The isolates were obtained from a previous study and subsequently made available for the present investigation. They were stored at −80°C in cryotubes containing 2 mL of BHI broth supplemented with 5% FBS and 20% glycerol until use. Before the experiments, the isolates were reconfirmed as *H. pylori* by Gram staining and biochemical tests, including catalase, oxidase, and urease tests. The complete list of the 48 clinical *H. pylori* isolates is provided in File S1.

### 2.4. Preparation of Plant Extracts

For each plant species, three separate 50 g portions of powdered plant material were weighed and individually macerated with 200 mL of dichloromethane, hexane, and methanol. Each mixture was maintained in a covered container for 72 h at room temperature with intermittent shaking, after which they were filtered through Whatman No. 1 filter paper. The residual plant material was re‐extracted with a fresh 200‐mL volume of the corresponding solvent for an additional 24 h to maximize extraction. The second filtrates were combined with their respective initial filtrates. The combined filtrates were concentrated under reduced pressure at 40°C using a rotary evaporator (Heidolph, Germany), and the resulting crude extracts were further dried in an oven at 30°C (BJPX‐Spring, BIOBASE). All crude extracts were stored at 4°C until use. Extraction yield was calculated using the formula (mass of dried crude extract/mass of dry plant material) × 100 [32]. The results are expressed as percentages.

### 2.5. Antimicrobial Activity of Plant Extracts on Planktonic Forms: MIC Determination

The plant extracts were further evaluated for their MICs using the INT‐based broth microdilution method described by Kouitcheu et al. with modifications [33]. The assay was performed in sterile U‐shaped 96‐well microtiter plates. Twofold serial dilutions of the plant extracts were prepared in BHI broth supplemented with 5% FBS resulting in final extract concentrations ranging from 0.9765 to 2000 *μ*g/mL. A 100‐*μ*L bacterial inoculum adjusted to a McFarland turbidity standard of 3.0 and prepared from a 48‐h culture grown on Columbia agar supplemented with 10% (*v*/*v*) lysed horse blood and 1% (*v*/*v*) Vitox was added to each well containing 100 *μ*L of the corresponding extract dilution. Each extract concentration was tested in three experimental runs using three technical replicates. Growth control wells containing culture medium and bacterial inoculum without plant extract, and sterility control wells containing culture medium without bacterial inoculum, were included. Levofloxacin and clarithromycin were used as reference antibiotics (positive control) at final concentration ranges of 0.0156–16 *μ*g/mL each. The plates were covered with sterile plate sealers and incubated at 37°C for 48 h under microaerophilic conditions. After incubation, 40 *μ*L of a 0.2‐mg/mL solution of INT was added to each well, followed by incubation at 37°C for an additional 30 min. Viable bacterial cells reduced the yellow INT dye to a pink‐colored product. The MIC was defined as the lowest concentration of the test sample that completely prevented the color change, indicating inhibition of bacterial metabolic activity. The total antibacterial activity (TAA) of each plant extract was determined by dividing the mass of the extract obtained from 1 g of dry plant material by its corresponding MIC value.

### 2.6. Antibiofilm Assay

#### 2.6.1. Biofilm Formation Assay

Quantitative assessment of biofilm biomass was performed using the crystal violet microtiter plate assay, according to the method described by Costa et al. [34] with some modifications. Briefly, 100 *μ*L of BHI broth supplemented with 2% FBS was dispensed into sterile, flat‐bottom 96‐well polystyrene microtiter plates, followed by the addition of 100 *μ*L of bacterial inoculum adjusted to a McFarland turbidity standard of 3.0 and prepared from a 48‐h culture grown on Columbia blood agar. Control wells containing culture medium supplemented with NAC at concentrations ranging from 2.5 to 10 mg/mL and wells containing culture medium alone were included as controls. The plates were covered with sterile plate sealers and incubated for 72 h at 37°C under microaerophilic conditions without shaking. After incubation, the wells were washed three times with PBS (pH 7.4) to remove nonadherent cells. The plates were allowed to dry at room temperature for 1 h, after which 200 *μ*L of 0.1% (*w*/*v*) crystal violet solution was added to each well and incubated for 15 min. The dye solution was then removed, and the wells were washed three times with 1 × PBS to remove excess stain. The attached dye was solubilized with 70% (*v*/*v*) ethanol, and 100 *μ*L of the resulting solution was transferred to a new sterile microtiter plate. Absorbance was measured at 590 nm using a microplate reader (Infinite M200, Tecan). Only isolates showing MIC values ≤ 100 *μ*g/mL for the evaluated plant extracts were selected for the subsequent biofilm experiments. Each selected isolate was tested in three technical replicates and the mean optical density (OD) was calculated. For interpretation of biofilm‐forming ability, the mean OD of each isolate was compared with the mean OD of the control according to the criteria described by Costa et al. [34]: OD (isolate) ≤ OD (control), nonbiofilm producing; OD (control) ≤ OD (isolate) ≤ 2 OD (control), weak producer; 2 OD (control) ≤ OD (isolate) ≤ 4 OD (control), moderate producer; and 4 OD (control) ≤ OD (isolate), strong producer.

#### 2.6.2. Evaluation of the Biofilm Inhibitory Activity of Plant Extracts

The ability of the plant extracts to inhibit biofilm formation was evaluated in sterile, flat‐bottom 96‐well polystyrene microtiter plates according to the method described by Hamayeli et al. [35] with some modifications. Briefly, 100 *μ*L of each plant extract prepared at six concentrations (8 × MIC, 4 × MIC, 2 × MIC, MIC, 1/2MIC, and 1/4MIC) in BHI broth supplemented with 2% FBS was dispensed into the test wells. Subsequently, 100 *μ*L of bacterial inoculum adjusted to a McFarland turbidity standard of 3.0 and prepared from a 48‐h culture grown on Columbia blood agar was added to each well. Four controls (200 *μ*L each) were included for each assay: wells containing culture medium with bacterial inoculum (biofilm control), extract containing culture medium (extract control), culture medium containing 5 mg/mL NAC and bacterial inoculum (positive control), and culture medium alone (sterility control). The plates were covered with sterile plate sealers and incubated for 72 h at 37°C under microaerophilic conditions without shaking. After incubation, the wells were washed three times with PBS (pH 7.4) to remove nonadherent cells. The remaining attached biomass was stained with 200 *μ*L of 0.1% (*w*/*v*) crystal violet solution for 15 min at room temperature. The stain was removed, and the wells were washed three times with 1 × PBS to remove unbound dye. The retained crystal violet was solubilized with 70% (*v*/*v*) ethanol, and the absorbance of the resulting solution was measured at 590 nm using a microplate reader (Infinite M200, Tecan). The experiment was performed in three independent runs, with each sample tested in three technical replicates per run. The mean absorbance was calculated for each run and the percentage inhibition of biofilm formation at each extract concentration was calculated according to the following formula [35, 36]:

%inhibition=OD biofilm control−OD sterility control−OD test−OD exctract controlOD biofilm control−OD sterility control×100.

#### 2.6.3. Eradication of Preformed Biofilm by Plant Extracts

Biofilm were first established in sterile, flat‐bottom 96‐well polystyrene microtiter plates by incubating 100 *μ*L of standardized bacterial inoculum adjusted to a McFarland turbidity standard of 3.0 under microaerophilic conditions at 37°C for 72 h without shaking. After incubation, the culture medium was carefully aspirated to remove planktonic cells, and 200 *μ*L of fresh BHI broth supplemented with 2% FBS and containing the plant extracts at concentrations of 8 × MIC, 4 × MIC, 2 × MIC, MIC, 1/2MIC, and 1/4MIC was added to the preformed biofilms. The same controls described for the biofilm inhibition assay were included. The plates were subsequently incubated for 48 h at 37°C under microaerophilic conditions without shaking. After treatment, the remaining biofilm biomass was quantified using the crystal violet staining procedure described above. The experiment was performed in three independent runs, with each sample tested in three technical replicates per run. The mean absorbance was calculated for each run and the percentage of biofilm eradication at each extract concentration was calculated using the formula described above.

### 2.7. Statistical Analysis

The extraction yields were not generated from independent replicate extractions. Thus, comparisons of extraction yields were limited on numerical comparison of values. MIC assays were performed within three experimental runs using three technical replicates, and the consensus definitive values were reported directly without statistical averaging. Biofilm inhibition and eradication assays were performed with three technical replicates and repeated across three independent biological replicates; data were expressed as mean ± standard deviation (SD) of the three independent experiments. Results from biofilm inhibition and eradication assays between the treated and control (positive control) were compared using one‐way analysis of variance (ANOVA), followed by Dunnett′s multiple comparison test. Statistical analysis was conducted using GraphPad Prism Version 8.0 software, and a *p* value of < 0.05 was considered statistically significant. *p* values were reported according to the significance levels generated by the software.

## 3. Results

### 3.1. Plant Extract Yields

The extraction yields of the five plant materials obtained with the three solvents are presented in Table 1. Methanol consistently produced the highest overall extraction yield across all plant materials (5/5, 100%), whereas hexane and dichloromethane produced the lowest yields in 3/5 (60%) and 2/5 (40%) of the plant materials, respectively. The highest individual extraction yields were obtained with the methanol extracts of *S. aromaticum* flower buds and *P. guajava* leaves, at 23.46% and 16.61%, respectively. In contrast, the lowest extraction yields were obtained with the dichloromethane and hexane extracts of *C. citratus* aerial parts, at 1.82% and 0.82%, respectively.

### 3.2. MIC Determination of Plant‐Extracts and Antibiotics on *H. pylori* Planktonic Forms

The antimicrobial activity of the 15 plant extracts against the 48 clinical *H. pylori* isolates is presented in File S1, whereas the results for isolates showing MIC values below 100 *μ*g/mL are summarized in Table 2. MIC values varied among the plant extracts and *H. pylori* isolates, ranging from 31.25 to > 2000 *μ*g/mL. The lowest MIC value (31.25 *μ*g/mL) was observed for the hexane extract of *Mangifera indica* (MILH) against isolate Hp185.

**Table 2:** Yields, minimum inhibitory concentration (MIC in *μ*g/mL), and total antibacterial activity (TAA in mL/g) of plant extracts exhibiting MIC < 100 *μ*g/mL against tested *H. pylori* isolates.

Plant extract	Yield %	Clinical isolates																	
		HpHG31(1)		Hp185		Hp137		Hp112		Hp142		HpHG31(2)		Hp00130		Hp123		Hp41	
		MIC	TAA	MIC	TAA	MIC	TAA	MIC	TAA	MIC	TAA	MIC	TAA	MIC	TAA	MIC	TAA	MIC	TAA
PGLD	5.4	> 2000	> 27	> 2000	> 27	> 2000	> 27	**62.5**	**864**	> 2000	> 27	2000	27	> 2000	> 27	> 2000	> 27	500	**108**
PGLH	10.4	**62.5**	**1595.1**	1000	**104**	> 2000	**> 52**	2000	**52**	> 2000	**> 52**	> 2000	**> 52**	> 2000	**> 52**	125	**832**	2000	**52**
MILH	3.2	1000	**32**	**31.25**	**1024**	2000	**16**	> 2000	> 16	**62.5**	**512**	> 2000	> 16	> 2000	> 16	**62.5**	**512**	> 2000	> 16
SAFM	23.46	250	938.4	2000	117.3	500	469.2	500	469.2	250	938.4	**62.5**	**3753.6**	250	938.4	1000	234.6	**62.5**	**3753.6**
MFFM	10.64	2000	53.2	> 2000	> 53.2	**62.5**	**1702.4**	1000	106.4	500	212.8	250	425.6	**62.5**	**1702.4**	2000	53.2	1000	106.4
CCAM	8.48	2000	42.4	**62.5**	**1356.8**	1000	84.8	> 2000	> 42.4	2000	42.4	2000	42.4	2000	42.4	> 2000	> 42.4	> 2000	> 42.4
LEV	NA	2	NA	0.125	NA	0.125	NA	0.5	NA	2	NA	0.05	NA	0.25	NA	4	NA	0.25	NA
CLA	NA	16	NA	8	NA	0.5	NA	0.0625	NA	16	NA	1	NA	16	NA	16	NA	1	NA

*Note:* Values in bold indicate MIC values < 100 *μ*g/mL and corresponding high TAA values.

Abbreviations: CCAM, methanol extract of *Cymbopogon citratus*; CLA, clarithromycin; LEV, levofloxacin; MFFM, methanol extract of *Musa* ‘False Horn’; MILH, hexane extract of *Mangifera indica*; NA, not applicable; PGLD, dichloromethane extract of *Psidium guajava*; PGLH, hexane extract of *Psidium guajava*; SAFM, methanol extract of *Syzygium aromaticum*; TAA, total antibacterial activity.

In the present study, an MIC ≤ 100 *μ*g/mL was used as the criterion for high antimicrobial activity of the plant extracts. Among the 15 extracts evaluated, six (40%) inhibited at least one *H. pylori* isolate at MIC values within the 31.25–62.5 *μ*g/mL range. The MILH showed high activity against 3/48 isolates (6.25%), with MIC values ranging from 31.25 to 62.5 *μ*g/mL. The methanol extracts of *S. aromaticum* and *Mu.* ‘False Horn’ each showed an MIC of 62.5 *μ*g/mL against 2/48 isolates (4.17%). The methanol extract of *Cymbopogon citratus* (CCAM) and the hexane and dichloromethane extract of *Psidium guajava* (PGLD) equally showed activity at this concentration each against 1/48 isolates (2.08%).

The TAA of the extracts with MIC values < 100 *μ*g/mL is presented in Table 2. The highest TAA values were observed for *S. aromaticum* (3753.6 mL/g), *Mu.* ‘False Horn’ (1702.4 mL/g), *P. guajava* (1595.1 mL/g), and *C. citratus* (1356.8 mL/g).

Regarding the reference antibiotics, MIC values ranged from 0.0625 to > 16 *μ*g/mL for levofloxacin and from 0.01563 to 16 *μ*g/mL for clarithromycin. MIC values ≤ 1 *μ*g/mL were observed for 31/48 isolates (64.58%) with levofloxacin and 15/48 isolates (31.25%) with clarithromycin. Using the predefined criterion of MIC ≤ 100 *μ*g/mL, 9/48 isolates (18.75%) showed high susceptibility to at least one plant extract. These isolates were HpHG31(1), Hp185, Hp137, Hp112, Hp142, HpHG31(2), Hp00130, Hp123, and Hp41. Among these nine isolates, Hp185 showed susceptibility to two plant extracts, namely the MILH and the CCAM, whereas the remaining isolates showed susceptibility to only one plant extract.

### 3.3. Biofilm Formation Assay

The growth of nine *H. pylori* isolates was inhibited by at least one of the evaluated plant extracts at MIC values < 100 *μ*g/mL. These isolates were HpHG31(1), Hp185, Hp137, Hp112, Hp142, HpHG31(2), Hp00130, Hp123, and Hp41. Their ability to form biofilms was subsequently evaluated, and the mean OD values measured at 590 nm are presented in Figure 1. Based on the predefined OD criteria, the isolates were classified as nonbiofilm producing, weak, moderate, or strong biofilm producers. Eight of the nine isolates (88.9%) produced biofilms. Among these, six (75.0%) were classified as strong producers (HpHG31(2), Hp00130, Hp123, Hp41, Hp112, and Hp185), one (HpHG31(1)) as a moderate producer, and one (Hp137) as a weak producer. Hp142 was classified as nonbiofilm producing and was therefore excluded from subsequent antibiofilm assays.

**Figure 1:**
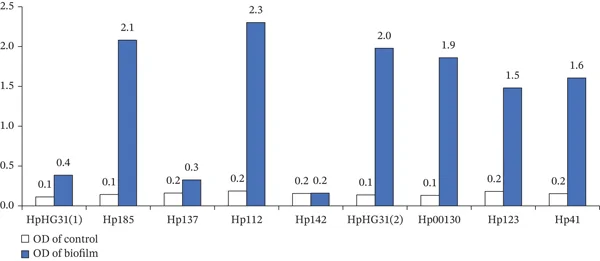
Optical density (DO) for biofilm producing isolates (590 nm).

### 3.4. Inhibition of Biofilm Formation: Antibiofilm Activity/Antiadhesion

The biofilm inhibition assay was performed using the plant extracts that exhibited MIC values ≤ 100 *μ*g/mL against the selected isolates. Six plant extracts were therefore evaluated for their ability to inhibit biofilm formation in the eight biofilm‐producing isolates.

According to the predefined criteria [36], percentage inhibition values between 0% and 100% indicated inhibition of biofilm formation, whereas inhibition ≥ 50% was considered good activity and inhibition between 0 %and 49% was considered poor activity. Negative percentage inhibition (< 0%) values indicated an enhancement of biofilm growth relative to the biofilm control.

Each extract was tested at six concentrations (8 × MIC, 4 × MIC, 2 × MIC, MIC, 1/2MIC, and 1/4MIC), and the minimum biofilm inhibition concentration (MBIC), defined here as the lowest concentration producing ≥ 50% inhibition of biofilm formation, was determined. The results were compared with those obtained with NAC (5 mg/mL), used as positive control. Each assay was conducted in three independent experiments, and the results are expressed as mean ± SD. The effects of the plant extracts on biofilm formation are presented in Table 3.

**Table 3:** Effect of plant‐extracts against biofilm formation by *H. pylori* isolates expressed as mean percentage biofilm inhibition (%).

Isolates	Extracts	Percentages of inhibition at different concentrations						NAC (5 mg/mL)
		8 × MIC	4 × MIC	2 × MIC	MIC	1/2MIC	1/4MIC	
HpHG31(1)	PGLH	$\begin{array}{c} 72.99\pm 0.06^{**} \end{array}$	$\begin{array}{c} 57.33\pm 0.07^{*} \end{array}$	$\begin{array}{c} 50.94\pm 0.01^{*} \end{array}$	$\begin{array}{c} 27.420.20\pm \end{array}$	$\begin{array}{c} -15.990.11\pm \end{array}$	$\begin{array}{c} -54.850.03\pm \end{array}$	$\begin{array}{c} 29.510.13\pm \end{array}$
Hp185	CCAM	$\begin{array}{c} 63.56\pm 0.0^{**} \end{array}$	$\begin{array}{c} 52.05\pm 0.06^{**} \end{array}$	$\begin{array}{c} 30.270.15\pm \end{array}$	$\begin{array}{c} 17.050.12\pm \end{array}$	$\begin{array}{c} 6.880.34\pm \end{array}$	$\begin{array}{c} -0.630.34\pm \end{array}$	$\begin{array}{c} 86.710.04\pm \end{array}$
	MILH	$\begin{array}{c} 58.42\pm 0.10^{**} \end{array}$	$\begin{array}{c} 50.24\pm 0.05^{**} \end{array}$	$\begin{array}{c} 44.910.01\pm \end{array}$	$\begin{array}{c} 37.990.14\pm \end{array}$	$\begin{array}{c} 33.100.39\pm \end{array}$	$\begin{array}{c} 29.600.28\pm \end{array}$	
Hp137	MFFM	$\begin{array}{c} 83.59\pm 0.02^{***} \end{array}$	$\begin{array}{c} 60.17\pm 0.09^{**} \end{array}$	$\begin{array}{c} 58.57\pm 0.00^{*} \end{array}$	$\begin{array}{c} 56.41\pm 0.00^{*} \end{array}$	$\begin{array}{c} -12.340.08\pm \end{array}$	$\begin{array}{c} -18.330.16\pm \end{array}$	$\begin{array}{c} 23.920.06\pm \end{array}$
Hp112	PGLD	$\begin{array}{c} 65.23\pm 0.00^{**} \end{array}$	$\begin{array}{c} 62.29\pm 0.01^{**} \end{array}$	$\begin{array}{c} 61.14\pm 0.00^{**} \end{array}$	$\begin{array}{c} 53.27\pm 0.24^{**} \end{array}$	$\begin{array}{c} 44.271.03\pm \end{array}$	$\begin{array}{c} 37.720.32\pm \end{array}$	$\begin{array}{c} 88.770.01\pm \end{array}$
HpHG31(2)	SAFM	$\begin{array}{c} 73.62\pm 0.04^{***} \end{array}$	$\begin{array}{c} 60.93\pm 0.05^{**} \end{array}$	$\begin{array}{c} 38.09\pm 0.00^{*} \end{array}$	$\begin{array}{c} 5.280.03\pm \end{array}$	$\begin{array}{c} -7.650.13\pm \end{array}$	$\begin{array}{c} -11.260.06\pm \end{array}$	$\begin{array}{c} 15.750.01\pm \end{array}$
Hp00130	MFFM	$\begin{array}{c} 50.27\pm 0.03^{***} \end{array}$	$\begin{array}{c} 21.14\pm 0.01^{**} \end{array}$	$\begin{array}{c} 6.070.03\pm \end{array}$	$\begin{array}{c} -1.550.00\pm \end{array}$	$\begin{array}{c} -16.780.01\pm \end{array}$	$\begin{array}{c} -19.860.01\pm \end{array}$	$\begin{array}{c} 9.460.01\pm \end{array}$
Hp123	MILH	$\begin{array}{c} 65.21\pm 1.20^{*} \end{array}$	$\begin{array}{c} 50.081.40\pm \end{array}$	$\begin{array}{c} 39.951.58\pm \end{array}$	$\begin{array}{c} 36.401.70\pm \end{array}$	$\begin{array}{c} 33.191.71\pm \end{array}$	$\begin{array}{c} 32.851.80\pm \end{array}$	$\begin{array}{c} 77.160.31\pm \end{array}$
Hp41	SAFM	$\begin{array}{c} 65.71\pm 0.08^{**} \end{array}$	$\begin{array}{c} 53.24\pm 0.02^{**} \end{array}$	$\begin{array}{c} 16.720.28\pm \end{array}$	$\begin{array}{c} 13.040.23\pm \end{array}$	$\begin{array}{c} 12.610.14\pm \end{array}$	$\begin{array}{c} 4.110.52\pm \end{array}$	$\begin{array}{c} 86.100.02\pm \end{array}$

*Note:* Values are expressed as mean ± SD of three independent experiments. Positive values indicate biofilm inhibition, whereas negative values indicate enhancement of biofilm formation. Values in bold indicate the biofilm inhibition percentages corresponding to the minimum biofilm inhibition concentration (MBIC), defined as the lowest concentration producing ≥ 50% biofilm inhibition. Asterisks indicate significant differences compared with NAC: ∗*p* < 0.05; ∗∗*p* < 0.01; ∗∗∗*p* < 0.001.

Abbreviations: CCAM, methanol extract of *Cymbopogon citratus*; MFFM, methanol extract of *Musa* ‘False Horn’; MILH, hexane extract of *Mangifera indica*; NAC, N‐acetyl‐L‐cysteine; PGLD, dichloromethane extract of *Psidium guajava*; PGLH, hexane extract of *Psidium guajava*; SAFM, methanol extract of *Syzygium aromaticum*.

The extracts showed isolate‐ and concentration‐dependent differences in their ability to inhibit biofilm formation. All six extracts exhibited good biofilm inhibitory activity against at least one isolate, although the MBIC varied among extracts and isolates. No extract achieved ≥ 50% inhibition at concentrations below its MIC. PGLD showed good activity at MIC against Hp112. Methanol extract of *Musa* ‘False Horn’ (MFFM) also achieved good inhibition at MIC against Hp137 and at 8 × MIC against Hp00130. Hexane extract of *Psidium guajava* (PGLH) showed good inhibition at 2 × MIC against HpHG31(1), whereas CCAM, MILH, and methanol extract of *Syzygium aromaticum* (SAFM) achieved good inhibition at 4 × MIC. Thus, PGLD and MFFM demonstrated the lowest MBIC among the evaluated extracts, achieving ≥ 50% biofilm inhibition at MIC against at least one isolate.

The positive control, NAC (5 mg/mL), also showed variable activity against the biofilm‐producing isolates. It produced ≥ 50% inhibition in four of the eight isolates (50.0%) and < 50% inhibition in the remaining four (50.0%). The lowest inhibition observed with NAC was 9.46*%* ± 0.01*%* against Hp00130. Among the isolates showing poor biofilm inhibition with NAC, MFFM, PGLH, SAFM, and MFFM achieved ≥ 50% inhibition at MIC, 2 × MIC, 4 × MIC, and 8 × MIC, respectively. Although Hp00130 showed the lowest response to NAC, its biofilm formation was inhibited by MFFM at 8 × MIC. The requirement for this relatively high extract concentration together with the low inhibition obtained with NAC, indicates that Hp00130 was the most difficult isolate to inhibit in terms of biofilm formation.

### 3.5. Eradication of Preformed Biofilms: Inhibition of Development of Preformed Biofilms—Assessment of Destruction of Biofilm Biomass

The potential of the plant extracts to eradicate 72‐h preformed biofilms is presented in Table 4. Each extract was evaluated at six concentrations (8 × MIC, 4 × MIC, 2 × MIC, MIC, 1/2MIC, and 1/4MIC), and the minimum biofilm eradication concentration (MBEC), defined as the lowest concentration producing ≥ 50% eradication of biofilm biomass, was determined. Each assay was performed in three independent experiments, and the results are expressed as mean ± SD. The values were compared with those obtained for the positive control (NAC, 5 mg/mL).

**Table 4:** Eradication percentages of preformed *Helicobacter pylori* biofilms by plant extracts.

Isolates	Extracts	Percentages of eradication at different concentrations						NAC (5 mg/mL)
		8 × MIC	4 × MIC	2 × MIC	MIC	1/2MIC	1/4MIC	
HpHG31(1)	PGLH	$\begin{array}{c} 58.80\pm 0.03^{*} \end{array}$	$\begin{array}{c} 53.43\pm 0.02^{*} \end{array}$	$\begin{array}{c} 52.45\pm 0.02^{*} \end{array}$	$\begin{array}{c} 31.930.01\pm \end{array}$	$\begin{array}{c} 17.900.05\pm \end{array}$	$\begin{array}{c} -6.990.10\pm \end{array}$	$\begin{array}{c} 26.290.09\pm \end{array}$
Hp185	CCAM	$\begin{array}{c} 52.49\pm 0.11^{**} \end{array}$	$\begin{array}{c} 52.29\pm 0.01^{**} \end{array}$	$\begin{array}{c} 48.750.01\pm \end{array}$	$\begin{array}{c} 32.910.05\pm \end{array}$	$\begin{array}{c} 31.220.00\pm \end{array}$	$\begin{array}{c} 16.250.01\pm \end{array}$	$\begin{array}{c} 15.610.03\pm \end{array}$
	MILH	$\begin{array}{c} 50.24\pm 0.05^{**} \end{array}$	$\begin{array}{c} 34.55\pm 0.14^{*} \end{array}$	$\begin{array}{c} 27.460.08\pm \end{array}$	$\begin{array}{c} 25.360.18\pm \end{array}$	$\begin{array}{c} 23.190.09\pm \end{array}$	$\begin{array}{c} 4.620.11\pm \end{array}$	
Hp137	MFFM	$\begin{array}{c} 68.11\pm 0.11^{**} \end{array}$	$\begin{array}{c} 58.16\pm 0.37^{*} \end{array}$	$\begin{array}{c} 53.26\pm 0.07^{*} \end{array}$	$\begin{array}{c} 44.430.11\pm \end{array}$	$\begin{array}{c} 47.520.24\pm \end{array}$	$\begin{array}{c} 34.790.14\pm \end{array}$	$\begin{array}{c} 89.830.09\pm \end{array}$
Hp112	PGLD	$\begin{array}{c} 37.080.07\pm \end{array}$	$\begin{array}{c} 15.690.03\pm \end{array}$	$\begin{array}{c} -10.810.06\pm \end{array}$	$\begin{array}{c} -16.210.07\pm \end{array}$	$\begin{array}{c} -18.580.05\pm \end{array}$	$\begin{array}{c} -24.400.09\pm \end{array}$	$\begin{array}{c} -29.860.21\pm \end{array}$
HpHG31(2)	SAFM	$\begin{array}{c} 57.81\pm 0.04^{*} \end{array}$	$\begin{array}{c} 55.62\pm 0.15^{*} \end{array}$	$\begin{array}{c} 31.770.51\pm \end{array}$	$\begin{array}{c} 27.160.03\pm \end{array}$	$\begin{array}{c} 11.660.02\pm \end{array}$	$\begin{array}{c} 9.450.24\pm \end{array}$	$\begin{array}{c} 62.720.04\pm \end{array}$
Hp00130	MFFM	$\begin{array}{c} 45.090.08\pm \end{array}$	$\begin{array}{c} 43.850.17\pm \end{array}$	$\begin{array}{c} 28.980.00\pm \end{array}$	$\begin{array}{c} 13.970.12\pm \end{array}$	$\begin{array}{c} 2.590.18\pm \end{array}$	$\begin{array}{c} -16.340.14\pm \end{array}$	$\begin{array}{c} -82.480.48\pm \end{array}$
Hp123	MILH	$\begin{array}{c} 15.860.17\pm \end{array}$	5.68 ± 0.02	$\begin{array}{c} -8.290.19\pm \end{array}$	$\begin{array}{c} -20.830.11\pm \end{array}$	$\begin{array}{c} -21.970.65\pm \end{array}$	$\begin{array}{c} -27.260.00\pm \end{array}$	$\begin{array}{c} 65.940.14\pm \end{array}$
Hp41	SAFM	$\begin{array}{c} 55.99\pm 0.00^{**} \end{array}$	$\begin{array}{c} 51.10\pm 0.06^{**} \end{array}$	$\begin{array}{c} 44.450.04\pm \end{array}$	$\begin{array}{c} 32.150.16\pm \end{array}$	$\begin{array}{c} 23.260.04\pm \end{array}$	$\begin{array}{c} 11.420.01\pm \end{array}$	$\begin{array}{c} 72.550.08\pm \end{array}$

*Note:* Values are expressed as mean ± SD of three independent experiments. Positive values indicate biofilm inhibition, whereas negative values indicate enhancement of biofilm formation. Values in bold biofilm eradication percentages corresponding to the minimum biofilm eradication concentration (MBAC), defined as the lowest concentration producing ≥ 50% biofilm eradication. Asterisks indicate significant differences compared with NAC: ∗*p* < 0.05; ∗∗*p* < 0.01; ∗∗∗*p* < 0.001.

Abbreviations: CCAM, methanol extract of *Cymbopogon citratus*; MFFM, methanol extract of *Musa* ‘False Horn’; MILH, hexane extract of *Mangifera indica*; NAC, N‐acetyl‐L‐cysteine; PGLD, dichloromethane extract of *Psidium guajava*; PGLH, hexane extract of *Psidium guajava*; SAFM, methanol extract of *Syzygium aromaticum*.

Extracts producing ≥ 50% eradication of preformed biofilms were considered to have good eradication activity. Whereas those producing < 50% but ≥ 0% eradication were considered to have poor activity [36]. Negative values indicated an increase in biofilm biomass relative to the biofilm control.

The plant extracts showed variable eradication activity against the preformed biofilms, with activity generally increasing with increasing extract concentration. Of the six extracts evaluated, five (PGLH, CCAM, MILH, MFFM, and SAFM) showed good eradication activity against at least one *H. pylori* isolate, whereas PGLD did not reach the 50% eradication threshold. Among the five active extracts, PGLH and MFFM achieved ≥ 50% eradication at 2 × MIC, CCAM and SAFM at 4 × MIC, and MILH at 8 × MIC. Thus, the lowest concentration associated with good biofilm eradication was 2 × MIC. At this concentration, PGLH and MFFM achieved eradication rates of 52.45*%* ± 0.02*%* against HpHG31(1) and 53.26*%* ± 0.07*%* against Hp137, respectively. Based on the corresponding MIC values, these activities were observed at 125 *μ*g/mL, representing the lowest effective eradication concentration among the extracts evaluated.

Some extracts exhibited both eradication and biofilm‐enhancing effects depending on the concentration and isolate tested. Against HpHG31(1), PGLH showed good eradication activity at 2 × MIC and higher concentrations, poor activity at MIC and 1/2MIC, and an increase in biofilm biomass at 1/4MIC (−6.99*%* ± 0.10*%*). PGLD showed poor eradication activity against Hp112 at 4 × MIC and 8 × MIC, whereas negative eradication values were observed from 1/4MIC to 2 × MIC indicating an increase in biofilm biomass at these concentrations. Similarly, MFFM showed poor eradication activity against Hp00130 from 1/2MIC to 8 × MIC, whereas 1/4MIC was associated with increased biofilm biomass. Against Hp123, MILH showed poor eradication activity at the concentrations tested, with a negative value at 2 × MIC (−8.29*%* ± 0.19*%*), indicating enhanced biofilm biomass at this concentration.

The positive control, NAC (5 mg/mL), showed good eradication activity against 4/8 of the evaluated biofilm‐producing isolates (50.0%; Hp137, HpHG31(2), Hp123, and Hp41), whereas poor eradication activity was observed against HpHG31(1) and Hp185 (25.0%). An increase in biofilm biomass was observed for Hp112 and Hp00130 (25.0%).

Among the isolates for which NAC was associated with increased biofilm biomass (Hp112 and Hp00130), none of the tested plant extracts achieved ≥ 50% eradication. PGLD showed poor activity against Hp112 at 4 × MIC and 8 × MIC, whereas MFFM showed poor activity against Hp00130 at concentrations from 1/2MIC to 8 × MIC. Hp112 and Hp00130 therefore appeared particularly difficult to eradicate, as none of the tested extracts achieved good eradication activity even at 8 × MIC. Hp123 was also relatively difficult to eradicate, although MILH produced measurable low‐level eradication across the concentrations tested.

### 3.6. Comparison of the Anti‐*Helicobacter* Activities of Plant Extracts Against Planktonic and Biofilm Forms

The anti‐*Helicobacter* activities of the plant extracts against the tested isolates in their planktonic and biofilm forms were compared. For this comparison, the MIC values obtained against planktonic cells were compared with the minimum concentrations required to achieve good inhibition of biofilm formation (MBIC) and good eradication of preformed biofilms (MBEC). The results are presented in Figure 2. The nine isolates showing susceptibility to at least one plant extract at MIC < 100 *μ*g/mL were considered for the planktonic comparison. For the biofilm assays, eight isolates were included, as Hp142 did not produce biofilm and was therefore excluded form future biofilm analysis.

**Figure 2:**
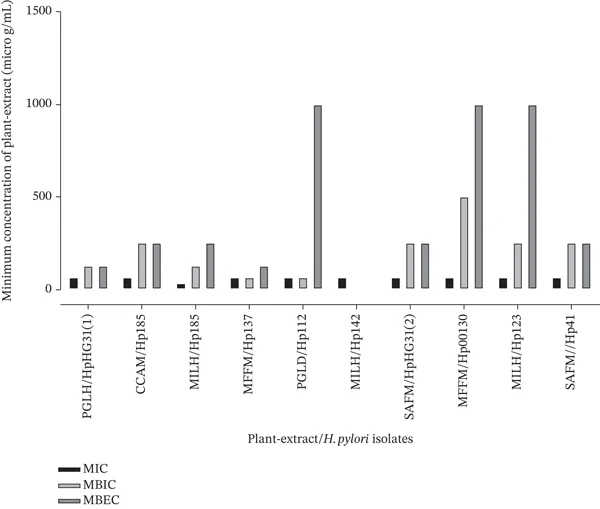
Minimum concentrations obtained by the plant‐extracts on both forms of the isolates. MIC, minimum inhibitory concentration; MBIC, minimum biofilm inhibition concentration; MBEC, minimum biofilm eradication concentration; PGLH, hexane extract of *Psidium guajava*; PGLD, dichloromethane extract of *Psidium guajava*; CCAM, methanol extract of *Cymbopogon citratus*; MILH, hexane extract of *Mangifera indica*; MFFM, methanol extract of *Musa* ‘False Horn’; SAFM, methanol extract of *Syzygium aromaticum.*

Overall, plant extracts were more active against planktonic cells than against biofilm‐associated cells. The lowest MIC values recorded against the planktonic forms ranged from 31.25 to 62.5 *μ*g/mL, whereas the concentrations required to achieve good inhibition of biofilm formation were generally higher, ranging from 62.5 to 500 *μ*g/mL. Similarly, the minimum concentrations required for good eradication of preformed biofilms ranged from 125 to > 500 *μ*g/mL. Thus, the concentrations required to inhibit biofilm formation and particularly to eradicate established biofilms were generally higher than those required to inhibit planktonic growth, indicating greater tolerance of the biofilm state to the tested plant extracts.

## 4. Discussion

The effectiveness of current therapeutic regimens against *H. pylori* remains limited, particularly in the context of increasing AMR and the bacterium′s ability to persist in biofilm communities. Biofilm formation may contribute to reduced antimicrobial susceptibility by providing a protective environment that limits antimicrobial penetration and promotes bacterial persistence. These characteristics can contribute to treatment failure and underscore the need for complementary approaches capable of targeting both planktonic and biofilm‐associated forms of *H. pylori* [5, 15]. In this context, the present study investigated the antibacterial and antibiofilm activities of extracts from five medicinal plants used in Cameroonian traditional medicine and cuisine.

In total, plant materials from five species were extracted using three solvents, and extraction yields varied according to the solvent used. Methanol produced the highest yield for all five plant materials whereas hexane and dichloromethane generally produced lower yields in three and two of the five plants, respectively. These differences may be related to solvent polarity, which influences the solubility and recovery of phytochemical constituents [37]. Methanol, being a polar solvent, can efficiently solubilize a broad range of polar and moderately polar constituents, including phenolic compounds, flavonoids, tannins, and other secondary metabolites [38]. However, extraction yield alone does not necessarily reflect antimicrobial potency, as biological activity depends on the nature, concentration, and interactions of the compounds recovered.

The antibacterial activity of the plant extracts varied substantially among the 48 clinical isolates, with MIC values ranging from 31.25 *μ*g/mL to values exceeding 2000 *μ*g/mL (File S1). Using the activity criterion adopted in this study (MIC ≤ 100 *μ*g/mL) [39], 40% of the evaluated extracts inhibited at least one clinical isolate within this concentration range. The hexane extract *Ma. indica* showed the lowest MIC values among the extracts tested, ranging from 31.25 to 62.5 *μ*g/mL. These values were lower than the MIC previously reported for an ethanol extract of *Ma. indica* (92.61 *μ*g/mL) [29], suggesting that the extraction solvent and/or plant material used may influence the recovery of antibacterial constituents. Previous studies have reported antibacterial activity of *Ma. indica* and have associated this activity with several classes of phytochemicals [29]. Nevertheless, such compounds should be regarded only as possible contributors to the activity observed in the present study because the extracts investigated here were not chemically characterized. The difference in activity between the extracts reported in different studies may reflect differences in solvent polarity, plant part, extraction procedure, geographical origin, or phytochemical composition. Other extracts also inhibited the clinical isolates at relatively low concentrations. The methanol extracts of *S. aromaticum* and *Mu.* ‘False Horn’ exhibited MIC values of 62.5 *μ*g/mL. Similarly, the CCAM and the hexane and PGLD also exhibited MIC values within the active range. These findings are considerably lower than the antibacterial activity observed by Ngnameko et al. [40] that reported MIC values of 50 mg/mL with leaf extracts of *P. guajava*. This difference may be related to variations in extraction procedures, plant part, bacterial strains, and experimental conditions. Similarly, Castillo‐Juárez et al. [31] reported an MIC of 31.1 *μ*g/mL for *C. citratus* leaf extracts, which is comparable with the low MIC values observed in the present study. The antibacterial activity observed with *Mu.* ‘False Horn’ peel extract is also consistent with previous reports highlighting the antimicrobial potential of plantain‐derived products [25, 27]. The activity of this extract may be associated with its diverse secondary metabolites, including flavonoids, tannins, alkaloids, glycosides, and terpenoids [41]. Such compounds have been reported to exert antimicrobial effects through several mechanisms including disruption of bacterial membrane integrity and interference with bacterial virulence [30]. However, these mechanisms were not directly investigated in the present study and should therefore be considered possible explanations rather than demonstrated mechanisms.

In addition, to evaluate the antibacterial potency, the TAA of the most active extracts was determined by integrating MIC values with extraction yields [42]. TAA represents an estimate of the volume (mL) to which an extract obtained from 1 g of plant material could be diluted while retaining antibacterial activity at its MIC. The highest TAA values were observed for *S. aromaticum*, *Mu.* ‘False Horn,’ *P. guajava*, and *C. citratus*, with values of 3753.6, 1702.4, 1595.1, and 1356.8 mL/g, respectively. The high TAA observed for *S. aromaticum* reflects the combined effect of its extraction yield and MIC, resulting in a greater effective volume of extract per gram of starting plant material. This finding supports the potential of *S. aromaticum* for further investigation and bioprospecting [42].

The relatively heterogeneous response of the clinical isolates is also noteworthy. Only nine of the 48 isolates showed susceptibility to at least one plant extract at MIC values ≤ 100 *μ*g/mL. The relatively limited susceptibility observed among the isolates may be related to the structural and physiological characteristics of *H. pylori*. As a gram‐negative bacterium, *H. pylori* possesses an outer membrane that can limit the penetration of antimicrobial compounds, whereas enzymatic mechanisms may also contribute to the modification or inactivation of bioactive substances [43]. However, these mechanisms were not investigated in the present study and therefore remain possible explanations for the observed susceptibility patterns. The differences observed among isolates exposed to the same extract may reflect strain‐to‐strain variation, although no genetic characterization or AMR profile was performed in the present study. Similarly, differences in activity among extracts tested against the same isolate may result from variations in the qualitative and quantitative composition of their bioactive constituents. These may arise from the extraction solvent, plant part examined, and plant species itself. The different mechanisms of action of phytochemicals may also contribute to differences in antibacterial activity [35].

The antibacterial activity of the plant extracts was also compared with those of reference antibiotics, levofloxacin, and clarithromycin. The observed MIC values of the antibiotics indicate reduced susceptibility among a proportion of the tested isolates, consistent with the AMR challenges reported for *H. pylori* in many emerging countries [13]. Although some plant extracts exhibited antibacterial activity comparable with those of the reference antibiotics under the conditions tested, direct equivalence should be interpreted cautiously. The antibiotics are purified compounds with established pharmacological properties, whereas the plant extracts were crude mixtures whose active constituents and concentrations were not determined. Interactions among these constituents may contribute to the observed antibacterial activity, either through additive or synergistic effects or through interactions that reduce the activity of individual compounds [44].

Bacteria embedded within biofilms are known to exhibit greater tolerance to antimicrobial agents than their planktonic counterparts [45]. In the present study, extracts showing high antibacterial activity (MIC < 100 *μ*g/mL) against planktonic cells were further evaluated for their ability to inhibit biofilm formation. Eight biofilm‐producing isolates were investigated, and the selected extracts were tested at concentrations ranging from 8 × MIC to 1/4MIC. All tested extracts inhibited at least 50% of biofilm formation in one or more isolates at one or more concentrations (Table 3).

Among the most active extracts, the PGLD and the MFFM showed good biofilm inhibition at their respective MICs against Hp112 and Hp137, respectively, whereas the PGLH achieved similar activity at 2 × MIC against HpHG31(1). These findings suggest that PGLD and MFFM were particularly promising for preventing biofilm formation because effective inhibition was achieved at concentrations equivalent to their planktonic MICs. Previous studies have also reported the ability of *P. guajava* extracts to interfere with bacterial adhesion and biofilm formation, including against oral pathogens such as *Streptococcus mutans* [46]. Other extracts, including CCAM, MILH, and SAFM, required higher concentrations to achieve comparable biofilm inhibition, indicating differences in antibiofilm potency among the extracts. Interestingly, MFFM also showed good inhibition against Hp00130 only at a substantially higher concentration, highlighting the isolate‐dependent nature of the antibiofilm response. The findings showed that antibacterial activity against planktonic cells did not necessarily translate into equivalent activity against biofilms.

Comparison with the positive control further demonstrated this variability. NAC at 5 mg/mL showed variable and, in several isolates, limited inhibitory activity against biofilm formation. Notably, its lowest inhibition was observed against Hp00130, whereas MFFM achieved good inhibition against this isolate at a higher concentration. Other extracts, including SAFM and PGLH, also showed good inhibitory activity against isolates that were poorly responsive to NAC, although at different concentrations. These observations suggest that the plant extracts may contain bioactive constituents capable of interfering with biofilm development and could therefore represent potential sources of antibiofilm agents [47–49]. Several mechanisms can be hypothesized based on the existing literature. Plant‐derived compounds may interfere with initial adhesion by altering physicochemical interactions between bacterial cells and surfaces, including electrostatic and hydrostatic interactions [50]. They may also influence nutrient availability and bacterial growth, which are important for biofilm development [51]. Interference with quorum‐sensing pathways that regulate bacterial communication and contribute to biofilm development has also been proposed as a possible mechanism of plant‐derived antibiofilm activity [24, 52]. However, these mechanisms were not directly investigated in the present study and therefore remain possible explanations for the observed inhibitory effects.

The extracts were also evaluated for their ability to eradicate established biofilms after 72 h of biofilm development. The findings showed that eradication of established biofilms generally required higher concentrations than those needed to inhibit biofilm formation, with the lowest MBEC observed at 125 *μ*g/mL for MFFM and PGLH. These extracts reduced biofilm biomass by 53.26% against Hp137 and 52.45% against HpHG31(1), respectively. These findings indicate that, although some extracts were able to reduce established biofilm biomass, their activity was dependent on both the extract and the bacterial isolate. This observation is consistent with previous reports describing the antibiofilm potential of plant‐derived compounds against bacterial pathogens [4, 53]. The activity observed with MFFM and PGLH further supports their potential as sources of compounds with antibiofilm activity, although the active constituents for these effects remain to be identified. However, the present study did not evaluate cytotoxicity of these concentrations in vivo; therefore, no conclusion can be drawn regarding the safety or therapeutic applicability. The potential application of these extracts into oral health care products, such as herbal mouthwashes for controlling oral *H. pylori* colonization also warrants caution. Previous studies have suggested that the oral cavity may act as an extragastric reservoir for *H. pylori* [54, 55], and that oral colonization may be associated with oral and gastric recurrence [56, 57]. Meanwhile, some studies have proposed the use of plant‐based mouthwashes as an important complementary strategy for improving eradication rates [58]. Although the present findings provide preliminary evidence that selected extracts can reduce *H. pylori* biofilm biomass in vitro, they do not establish efficacy as mouthwash ingredients or demonstrate that such an application would reduce gastric reinfection.

An important finding requiring particular consideration was the increase in biofilm biomass observed with some extracts at specific concentrations after 72 h of biofilm formation. This effect was observed with hexane and PGLD, MFFM, and MILH, although the response varied according to the extract, concentration, and isolate. Such concentration‐dependent enhancement has also been reported for some natural products, suggesting that plant‐derived compounds may exert different effects on bacterial growth and biofilm development depending on their concentration and composition [59]. One possible explanation is that, at subinhibitory concentrations, certain phytochemicals may modify bacterial adhesion or the biofilm environment rather than exerting a direct inhibitory effect. For example, plant lectins and other compounds capable of interacting with bacterial surfaces may partially influence adhesion and biofilm development [60]. Comparable observations have been reported by Sandasi et al. [51] in their evaluation of plant‐derived products against *Listeria monocytogenes*. The ability of *H. pylori* to form biofilms is associated with the production of an extracellular polymeric matrix, which contributes to the stability and protection of the bacterial community and can reduce antimicrobial penetration [61].

Consistent with this protective nature of biofilms, Hp112, Hp00130, and Hp123 remained difficult to eradicate even at the highest concentrations tested. This isolate dependent response further suggests that differences in biofilm characteristics may influence the susceptibility of established *H. pylori* biofilms to plant‐derived extracts [14, 45].

Although the extracts demonstrated antibiofilm activity, their ability to eradicate established biofilms was generally lower than their capacity to inhibit biofilm formation. This finding suggests that preventing biofilm development may be more readily achievable than eliminating mature biofilms. Such observation is consistent with previous studies showing that bacteria embedded within biofilms exhibit increased tolerance to antimicrobial agents, allowing infections to persist on both biological and inert surfaces [62]. Similar findings have been reported by Mohsenipour and Hassanshahian [63] in their evaluation of *Allium sativum* extract against *Escherichia coli*. Other studies have likewise shown that lower concentrations of plant extracts may be sufficient to inhibit biofilm formation, whereas higher concentrations are required to disrupt established biofilms [64, 65]. This difference may be related to several characteristics of mature biofilms, including limited antimicrobial penetration through the extracellular polymeric matrix and the activity of efflux systems that reduce intracellular antimicrobial accumulation [6].

The present study has some limitations that need to be acknowledged. The characterization of the active constituents of the extracts investigated and the specific mechanisms underlying their activity were not determined. Furthermore, the crystal violet assay used measures total biofilm biomass and does not distinguish viable from nonviable cells. Consequently, the observed reduction in biofilm biomass cannot itself establish bacterial killing or loss of cellular viability. Complementary approaches, such as viable‐cell enumeration, live‐dead staining, or resazurin‐based metabolic assays, would therefore be useful to better characterize the effects of these extracts on viable *H. pylori* cells within biofilms.

## 5. Conclusion

Among the 15 plant extracts evaluated, six demonstrated notable antibacterial activity against planktonic *H. pylori* isolates, with MIC values ranging from 31.25 to 62.50 *μ*g/mL. The MILH leaves showed the highest antibacterial activity against 3/48 (6.25%) of the tested isolates. Regarding antibiofilm activity, the MFFM fruit peelings and the PGLD leaves demonstrated the most promising inhibitory activity, with MBIC values of 62.5 *μ*g/mL, whereas the PGLH and the MFFM showed the lowest MBEC values (125 *μ*g/mL) for pre‐established biofilms. Overall, the findings indicate that some plant extracts possess activity against both planktonic and biofilm‐associated *H. pylori*, although higher concentrations were generally required against biofilms, particularly pre‐established biofilms. These findings support further investigation of the active constituents and mechanisms underlying the observed antibacterial and antibiofilm effects.

**Nomenclature**
BHI: brain heart infusion
CCAM: methanol extract of *Cymbopogon citratus* aerial parts
DCM: dichloromethane
FBS: fetal bovine serum
Hex: hexane
HNC: National Herbarium Cameroon
INT: *ρ*‐iodonitrotetrazolium chloride
MBEC: minimum biofilm eradication concentration
MBIC: minimum biofilm inhibition concentration
MeOH: methanol
MFFM: methanol extract of *Musa* ‘False Horn’ fruit peelings
MIC: minimum inhibitory concentration
MILH: hexane extract of *Mangifera indica* leaves
NAC: N‐acetyl‐L‐cysteine
OD: optical density
PBS: phosphate‐buffered saline
PGLD: dichloromethane extract of *Psidium guajava* leaves
PGLH: hexane extract of *Psidium guajava* leaves
SAFM: methanol extract of *Syzygium aromaticum* flower buds
SD: standard deviation
TAA: total antibacterial activity

## Author Contributions

K.M.L.B. conceived the study, designed the experiments, supervised the work, drafted, and submitted the manuscript. K.N.C.A. carried out the experiments and data analysis and revised the manuscript. K.M.L.B. participated in plant collection and provided the facilities for the study.

## Funding

No funding was received for this manuscript.

## Disclosure

All authors read and approved the final manuscript.

## Ethics Statement

The authors have nothing to report.

## Consent

The authors have nothing to report.

## Conflicts of Interest

The authors declare no conflicts of interest.

## Supporting information


**Supporting Information** Additional supporting information can be found online in the Supporting Information section. File S1: Minimum inhibitory concentration values (in *μ*g/mL) of the tested plant extracts and reference antibiotics against the 48 clinical *Helicobacter pylori* isolates.

## Data Availability

The data that support the findings of this study are available from the corresponding author upon reasonable request.
