# Supplementary material for: Anti‐Helicobacter pylori Activity of Musa ‘False Horn’ (Musaceae), Syzygium aromaticum (Myrtaceae), Psidium guajava (Myrtaceae), Mangifera indica (Anacardiaceae), and Cymbopogon citratus (Poaceae) and Their Potential to Prevent and Eradicate H. pylori Biofilms
Source: Biomed Res Int. 2026 Oct 28;2026:1930477. doi: 10.1155/bmri/1930477 (PMC13715488; doi:10.1155/bmri/1930477)
Supplement: Supplementary file 1 [file BMRI-2026-1930477-s001.docx]

**Supplementary File 1:** MIC values of plant-extracts used against the 48 clinical *Helicobacter pylori* isolates tested.

| **Isolates** | ***Psidium guayava*** | | | ***Mangifera indica*** | | | ***Syzygium aromaticum*** | | | ***Musa* ‘False Horn’** | | | ***Cymbopogon citratus*** | | | **LEV** | **CLA** |
| --- | --- | --- | --- | --- | --- | --- | --- | --- | --- | --- | --- | --- | --- | --- | --- | --- | --- |
|  | **MeOH** | **DCM** | **HEX** | **MeOH** | **DCM** | **HEX** | **MeOH** | **DCM** | **HEX** | **MeOH** | **DCM** | **HEX** | **MeOH** | **DCM** | **HEX** |  |  |
| Hp24 | >2000 | >2000 | 1000 | 2000 | >2000 | 2000 | 500 | 2000 | 1000 | 500 | 1000 | 2000 | 2000 | >2000 | 1000 | 0.5 | 8 |
| Hp97 | >2000 | >2000 | 1000 | >2000 | >2000 | 500 | 1000 | 2000 | 2000 | >2000 | >2000 | >2000 | >2000 | >2000 | >2000 | 1 | 16 |
| Hp148 | >2000 | >2000 | >2000 | 2000 | >2000 | >2000 | 1000 | >2000 | 2000 | 2000 | 2000 | >2000 | >2000 | >2000 | >2000 | 0.5 | 0.0625 |
| HpHG20 | >2000 | >2000 | 250 | 1000 | >2000 | 250 | >2000 | >2000 | 1000 | 2000 | 1000 | 2000 | >2000 | >2000 | >2000 | 0.5 | 16 |
| Hp136 | >2000 | >2000 | >2000 | 2000 | >2000 | >2000 | 1000 | 2000 | 250 | 1000 | 2000 | 2000 | >2000 | >2000 | >2000 | 2 | 16 |
| Hp99 | >2000 | 2000 | 1000 | 2000 | >2000 | >2000 | 500 | 1000 | 500 | 1000 | 2000 | >2000 | 1000 | >2000 | >2000 | 0.125 | 4 |
| HpHG54 | >2000 | >2000 | 2000 | 250 | >2000 | 250 | 1000 | >2000 | 1000 | 500 | >2000 | >2000 | 1000 | >2000 | >2000 | 0.25 | 16 |
| Hp05 | >2000 | >2000 | >2000 | 1000 | >2000 | 250 | 1000 | 1000 | 2000 | >2000 | 1000 | 2000 | 2000 | >2000 | >2000 | 2 | 8 |
| Hp132 | 1000 | 2000 | 125 | 1000 | >2000 | >2000 | 2000 | >2000 | >2000 | 1000 | >2000 | >2000 | 250 | >2000 | >2000 | 0.25 | 4 |
| HpHG31 (1) | 2000 | >2000 | **62.5** | 2000 | >2000 | 1000 | 250 | 250 | 500 | 2000 | 2000 | 1000 | 2000 | >2000 | >2000 | 2 | 16 |
| Hp185 | 2000 | >2000 | 1000 | 1000 | >2000 | **31.25** | 2000 | >2000 | >2000 | >2000 | >2000 | 2000 | **62.5** | >2000 | >2000 | 0.125 | 8 |
| Hp143 | 2000 | >2000 | >2000 | 1000 | 2000 | >2000 | 500 | >2000 | 1000 | 2000 | >2000 | >2000 | >2000 | >2000 | >2000 | 4 | 16 |
| Hp129 | 2000 | >2000 | >2000 | 2000 | >2000 | >2000 | 250 | >2000 | 500 | 250 | 500 | 1000 | 2000 | >2000 | >2000 | 2 | 16 |
| Hp137 | 2000 | >2000 | >2000 | 1000 | >2000 | 2000 | 500 | 1000 | 1000 | **62.5** | 1000 | 1000 | 1000 | >2000 | >2000 | 0.125 | 0.5 |
| Hp131 | 1000 | 2000 | >2000 | 1000 | >2000 | >2000 | 1000 | >2000 | >2000 | 2000 | >2000 | >2000 | 2000 | >2000 | >2000 | 0.5 | 0.5 |
| Hp29 | 2000 | >2000 | 1000 | 1000 | >2000 | 1000 | 500 | >2000 | 2000 | 500 | 2000 | 2000 | >2000 | >2000 | >2000 | 2 | 16 |
| HpHG050 | 2000 | >2000 | 2000 | 2000 | >2000 | 2000 | 125 | 250 | 125 | 250 | 500 | 1000 | 2000 | >2000 | >2000 | 2 | 16 |
| Hp112 | 2000 | **62.5** | 2000 | 1000 | 1000 | >2000 | 500 | >2000 | 1000 | 1000 | 2000 | 2000 | >2000 | >2000 | >2000 | 0.5 | 0.0625 |
| Hp142 | 2000 | >2000 | >2000 | 2000 | >2000 | **62.5** | 250 | >2000 | 1000 | 500 | 1000 | 2000 | 2000 | >2000 | >2000 | 2 | 16 |
| HpHG020 | 1000 | >2000 | >2000 | 1000 | >2000 | >2000 | 500 | 2000 | 2000 | 500 | 1000 | >2000 | >2000 | >2000 | >2000 | 0.5 | 16 |
| Hp0064 | 1000 | >2000 | >2000 | 125 | 1000 | >2000 | 125 | 1000 | 500 | 500 | 1000 | 1000 | >2000 | >2000 | >2000 | 0.5 | 16 |
| Hp111 | 2000 | 2000 | >2000 | 2000 | >2000 | >2000 | 1000 | >2000 | >2000 | 2000 | >2000 | >2000 | 2000 | >2000 | >2000 | 2 | 1 |
| HpHG31(2) | 250 | 2000 | >2000 | 250 | 2000 | >2000 | **62.5** | 250 | 500 | 250 | 1000 | 1000 | 2000 | >2000 | >2000 | 0.5 | 1 |
| HpHG42 | 1000 | >2000 | >2000 | 1000 | >2000 | >2000 | 1000 | 2000 | 2000 | 2000 | 2000 | 2000 | >2000 | >2000 | >2000 | 0.0625 | 0.125 |
| Hp00146 | 1000 | >2000 | >2000 | 1000 | 1000 | 1000 | 500 | >2000 | 1000 | 1000 | 2000 | 2000 | >2000 | >2000 | >2000 | 1 | 16 |
| HpHG34 | 500 | >2000 | >2000 | 500 | >2000 | 2000 | 500 | >2000 | 500 | 10000 | 1000 | 1000 | 2000 | >2000 | >2000 | 0.25 | 16 |
| HpHG41 | 2000 | >2000 | 2000 | 500 | >2000 | >2000 | 500 | 2000 | 1000 | 1000 | 1000 | 2000 | 2000 | >2000 | >2000 | 0.125 | 16 |
| Hp00130 | >2000 | >2000 | >2000 | 1000 | >2000 | >2000 | 250 | 1000 | 500 | **62.5** | 500 | 250 | 2000 | >2000 | 2000 | 0.25 | 16 |
| Hp0057 | >2000 | 2000 | 2000 | 250 | >2000 | >2000 | 2000 | >2000 | >2000 | >2000 | >2000 | >2000 | >2000 | >2000 | >2000 | 0.125 | 16 |
| Hp147 | 500 | >2000 | 2000 | 250 | >2000 | 1000 | 125 | >2000 | 1000 | 250 | 1000 | 2000 | >2000 | >2000 | 500 | 0.5 | 0.0625 |
| Hp81 | 500 | >2000 | 2000 | 250 | >2000 | 250 | 250 | 125 | 250 | 1000 | 500 | 2000 | >2000 | >2000 | >2000 | 0.5 | 16 |
| Hp98 | 2000 | >2000 | >2000 | 1000 | >2000 | 250 | 500 | 2000 | 2000 | >2000 | >2000 | >2000 | >2000 | >2000 | >2000 | 0.0625 | 1 |
| Hp83 | 2000 | >2000 | 1000 | 2000 | 250 | 2000 | 1000 | >2000 | >2000 | >2000 | 2000 | >2000 | >2000 | >2000 | >2000 | 0.25 | 16 |
| Hp123 | 2000 | >2000 | 125 | >2000 | >2000 | **62.5** | 1000 | 2000 | 1000 | 2000 | 1000 | 2000 | >2000 | >2000 | >2000 | 4 | 16 |
| Hp0091 | 2000 | >2000 | >2000 | 2000 | >2000 | 1000 | 2000 | >2000 | >2000 | >2000 | >2000 | >2000 | >2000 | >2000 | >2000 | 0.0625 | 0.01563 |
| Hp41 | 500 | 500 | 2000 | 250 | 250 | >2000 | **62.5** | 250 | 2000 | 1000 | 1000 | 1000 | >2000 | 2000 | >2000 | 0.25 | 1 |
| Hp135 | 500 | >2000 | >2000 | 500 | >2000 | 1000 | 250 | >2000 | 1000 | 2000 | 500 | 2000 | >2000 | >2000 | >2000 | 4 | 1 |
| HpHG22 | 500 | 1000 | 2000 | 2000 | >2000 | 1000 | 1000 | 2000 | >2000 | >2000 | >2000 | >2000 | >2000 | >2000 | >2000 | 0.5 | 4 |
| Hp145 | 1000 | >2000 | >2000 | 2000 | >2000 | >2000 | >2000 | >2000 | >2000 | >2000 | >2000 | 2000 | >2000 | >2000 | >2000 | 0.5 | 0.0625 |
| Hp66 | 2000 | >2000 | >2000 | 1000 | >2000 | >2000 | >2000 | >2000 | >2000 | >2000 | >2000 | >2000 | >2000 | >2000 | >2000 | 0.0625 | 0.125 |
| HpHG41(7) | >2000 | >2000 | >2000 | >2000 | >2000 | >2000 | >2000 | >2000 | >2000 | >2000 | >2000 | >2000 | >2000 | >2000 | 1000 | >16 | 16 |
| Hp138 | >2000 | >2000 | >2000 | 500 | >2000 | >2000 | >2000 | >2000 | >2000 | >2000 | >2000 | >2000 | 2000 | >2000 | >2000 | >16 | 8 |
| HpHGD031 | 2000 | >2000 | >2000 | 500 | >2000 | >2000 | 2000 | >2000 | >2000 | >2000 | >2000 | >2000 | 2000 | >2000 | >2000 | 2 | 16 |
| Hp125 | 2000 | >2000 | >2000 | 2000 | >2000 | 1000 | 2000 | >2000 | >2000 | >2000 | >2000 | >2000 | >2000 | >2000 | >2000 | 16 | 16 |
| Hp140 | 2000 | >2000 | 2000 | 1000 | >2000 | >2000 | 2000 | 2000 | >2000 | 2000 | 1000 | 2000 | >2000 | >2000 | >2000 | >16 | 16 |
| Hp00127 | 2000 | >2000 | 1000 | 2000 | >2000 | 2000 | 2000 | >2000 | >2000 | >2000 | >2000 | 2000 | >2000 | >2000 | >2000 | 0.5 | 16 |
| Hp13 | 2000 | >2000 | >2000 | 2000 | >2000 | >2000 | 2000 | >2000 | >2000 | >2000 | 2000 | 2000 | 2000 | >2000 | >2000 | 0.5 | 4 |
| Hp0055 | 1000 | 1000 | >2000 | 500 | >2000 | >2000 | 2000 | >2000 | >2000 | 2000 | >2000 | >2000 | >2000 | >2000 | >2000 | 4 | 0.03125 |

DCM: Dichloromethane, Hex: Hexane, MeOH: Methanol. LEV: Levofloxacin, CLA: Clarithromycin. MIC values in bold indicate high anti-*Helicobacter* *pylori* activities exhibited by plant-extracts.
